# Discontinuation rates in clinical trials in musculoskeletal pain: meta-analysis from etoricoxib clinical trial reports

**DOI:** 10.1186/ar2422

**Published:** 2008-05-08

**Authors:** R Andrew Moore, Sheena Derry, Henry J McQuay

**Affiliations:** 1 Pain Research and Nuffield Department of Anaesthetics, University of Oxford, Oxford Radcliffe Hospital, The Churchill, Oxford OX3 7LJ, UK

## Abstract

**Introduction:**

Patient adherence to therapy in clinical practice is often low, and the difference between efficacy measured in clinical trials and effectiveness in clinical practice is probably a function of discontinuation of therapy because of lack of efficacy or because of unmanageable or intolerable adverse events. Discontinuation is frequently measured in clinical trials but is not usually described in detail in published reports, often because of limitations in the size of publications. By contrast, company clinical trial reports include much more detail.

**Methods:**

We examined company clinical trial reports of trials involving etoricoxib in four musculoskeletal conditions: osteoarthritis, rheumatoid arthritis, chronic low back pain and ankylosing spondylitis. Information was available from 18 randomized trials (10,143 patients) lasting 4 to 12 weeks (one 4 weeks, three 6 weeks, one 8 weeks and seven 12 weeks) and from three trials with a mean duration of about 80 weeks (34,695 patients). These clinical trial reports contain over 73,000 pages of information.

**Results:**

Over 12 weeks, lack of efficacy and adverse event discontinuations were similar between osteoarthritis, rheumatoid arthritis and back pain, with lack of efficacy discontinuation rates some three times higher than for adverse events. All-cause and lack of efficacy discontinuations were lower with etoricoxib (all doses combined) and traditional nonselective nonsteroidal anti-inflammatory drugs (NSAIDs) than with placebo, although NSAIDs produced higher rates of clinical adverse events and gastrointestinal discontinuations than did placebo. Etoricoxib had fewer discontinuations than NSAIDs for lack of efficacy, clinical adverse events, and laboratory and gastrointestinal adverse events, but with more discontinuations because of hypertension and oedema. Comparison with two similar meta-analyses of other cyclo-oxygenase-2 selective inhibitors (more than 80,000 patients in total) revealed consistency between analyses.

**Conclusion:**

Examining discontinuation data from clinical trials, even when the numbers of patients are very large, does not necessarily predict what will happen in the real world, where clinical effectiveness may differ from clinical efficacy assessed in trials. Data from these analyses appears to agree with findings from real world practice.

## Introduction

Clinical trials most often measure efficacy – the ability of an intervention to produce the desired result. They tend not to measure effectiveness, which are the actual results found in clinical practice – a product of efficacy and adherence to therapy. The difference between trial efficacy and clinical effectiveness can be ascribed to the tendency of patients not to adhere to therapy, especially in the longer term. Discontinuation of therapy is most often due to lack of efficacy or unmanageable or intolerable adverse events, or both.

Patient adherence to therapy is known to be low, especially where therapy is prophylactic or does not deliver rapid symptomatic relief. In the USA it is estimated that only about 50% of patients continue statin therapy at 6 months, and 30% to 40% at 1 year [1]. In the UK 50% of patients prescribed low-dose aspirin have discontinued within a year [2]. Where benefit is greater and more tangible, adherence is likely to be higher, even if adverse events are common. Thus, among renal transplant patients, only 15% were found to be nonadherent to immunosuppressants using stringent criteria [3].

Adverse events are a major concern to patients. A Dutch study [4] found that half of 232 chronic prescriptions for long-term medicines were not refilled over 3 months, with adverse events being a major reason. Talking to patients about adverse events can help, but it may well depend on how the adverse event rates are described. In a randomized trial conducted in 120 patients given information about the adverse events associated with the medicine [5], it was found that the patients were more likely to be compliant, and had less fear, when they were presented with information about adverse events in percentage terms rather than in words.

Physician adherence to guidelines is also known to be low. Prescribing of nonsteroidal anti-inflammatory drug (NSAID) plus gastroprotective agent or cyclo-oxygenase-2 inhibitor (coxib) was identified in only 26% of patients with at least one gastrointestinal risk factor in a large systematic review of studies published since 2000 [6]. More or better professional education can improve matters, as with antihypertensive prescribing in Canada [7,8].

Few patients appear able to take oral NSAIDs prescribed for chronic musculoskeletal problems for a long time. For instance, only 15% to 20% of those started on a study NSAID were still using the same drug at the end of 12 months in an observational study conducted in Seattle [9], whereas in a telephone survey [10], also conducted in the USA, drug continuation beyond 24 months was reported by 33% of patients for paracetamol (acetaminophen), 21% for ibuprofen, 17% for naproxen and 19% for diclofenac. Most studies are too small to measure differences in continuation rates between NSAIDs adequately, although recent larger studies have consistently observed longer continuation rates for coxibs over traditional nonselective NSAIDs [11,12].

Company clinical trial reports do not suffer from the problems of selective reporting in published papers that results from strict word limitations. Although efficacy in published studies has in the past been poorly presented [13], adverse event information is even more poorly presented in published papers [14]. Both, but particularly adverse events, may be more clearly presented in company clinical trial reports. Company clinical trial reports potentially provide an ideal source of information for systematic review and meta-analysis, particularly on discontinuation rates for different causes [15,16].

Meta-analysis of discontinuation rates in randomized trials of drug therapy has been undertaken infrequently. In psychiatry, studies in schizophrenia [17] and depression [18,19] have sought to differentiate drugs by discontinuation rates, as have studies in *Helicobacter *treatment [20] or hypertension [21]. Discontinuation rates have been included in previous meta-analyses of clinical trials of analgesics in musculoskeletal pain [15,16].

In this report we examine discontinuation rates in randomized trials of etoricoxib in chronic musculoskeletal conditions. Our prior hypotheses were as follows. First, discontinuation rates would differ between osteoarthritis (OA), rheumatoid arthritis (RA) and other musculoskeletal conditions for lack of efficacy, but probably not because of adverse events. Second, discontinuation rates would differ between shorter and longer trials. Third, discontinuations because of gastrointestinal adverse events would be lower with etoricoxib than with traditional NSAIDs. Finally, higher doses of etoricoxib would produce lower rates of lack of efficacy discontinuations and higher rates of adverse event discontinuations.

## Materials and methods

The data for this meta-analysis were contained in clinical trial reports of 18 randomized trials of etoricoxib lasting 4 to 12 weeks, and three trials with mean duration of more than 1 year. Merck Research Laboratories (Rahway, NJ, USA) provided clinical trial reports in the form of PDF (19 trials) or Word (two trials) documents. No additional literature searching was conducted.

Information from the clinical trial reports was tabulated, including diagnosis and trial entry criteria, demographic information, drugs, dose and duration of treatment. Patients who were randomized and received at least one dose of drug were considered the intention-to-treat population. Also extracted were the following outcomes: all-cause discontinuations; discontinuations due to lack of efficacy; discontinuations due to a clinical adverse event; discontinuations due to a laboratory adverse event (hepatic, renal, or haematological); and discontinuations for specific reasons, namely gastrointestinal adverse event, oedema, hypertension, or a serious adverse event.

In addition, information about the time course of discontinuations was sought. Where this information was in graphical form, printed versions were used to calculate the number of patients remaining after discontinuation for lack of efficacy or adverse event. Extrapolation was not done where the scale of graphs precluded accurate estimation.

The nomenclature for discontinuation does not appear to be fixed. Although discontinuations for a particular cause might be described as a percentage of the total (100*x*/*n*, where *x *is the number discontinued and *n *the total number of patients), it is common for discontinuations to be described as a continuation (100 - 100*x*/*n*) to produce a description of survival on treatment. This has previously been done for NSAIDs [9], and we chose to follow this example to describe discontinuation over the first 12 weeks of treatment.

Trial reporting quality and validity were examined using two Oxford scales [22,23]. Relative risk with 95% confidence interval was calculated using the fixed effects model [24], and was considered to be statistically significant when the 95% confidence interval did not include 1. We calculated the number needed to treat to prevent an event (NNTp) or cause an event (NNH), with a 95% confidence interval, from the sum of all events and patients for treatment and placebo groups [25]. Heterogeneity tests were not used because they have previously been shown to be unhelpful, although homogeneity was examined visually [26,27]. Publication bias was not assessed using funnel plots because these tests have been shown to be unhelpful [28,29]. QUOROM (Quality Of Reporting Of Meta-analyses) guidelines were followed where appropriate [30].

## Results

### Trials

Information was available for 18 randomized trials (10,143 patients) lasting 4 to 12 weeks (one 4 weeks, three 6 weeks, one 8 weeks and seven 12 weeks) and three trials (34,695 patients) with a mean duration about 80 weeks. These clinical trial reports contained over 73,000 pages of information, mainly in the form of PDF documents. These were comprehensive documents detailing methods and results, with many tables and figures. Five trials were conducted completely or mainly in patients with RA, 12 completely or mainly in patients with OA, three in chronic low back pain, and one in ankylosing spondyitis. Details of trials and outcomes are presented in Additional file 1. All trials scored 5 out of 5 on a validated quality scale and 16 out of 16 on a validity scoring scale.

Sixteen of the 4-week to 12-week trials included a placebo control group (2,336 patients receiving placebo), in which up to two 325 mg tablets of paracetamol (acetaminophen) were allowed as rescue medication to a maximum of four times a day for short periods. Phase III and IV trials studied daily doses of etoricoxib of 30 mg and 60 mg in OA patients; the 90 mg daily dose, representing 1.5 times the maximum recommended daily dose, was also studied in OA patients in two trials (061 EDGE and 066 MEDAL trials). The daily dose of 90 mg was assessed in patients with RA. Daily doses of 60 mg and 90 mg (1.5 times the maximum recommended daily dose) were assessed in patients with chronic low back pain; daily doses of 90 mg and 120 mg (1.33 times the maximum recommended daily dose) were assessed in patients with ankylosing spondylitis. The comparator cyclo-oxygenase-2 selective inhibitor was celecoxib 200 mg or 400 mg daily. Comparator NSAIDs were naproxen 1,000 mg daily, diclofenac 150 mg daily and ibuprofen 2,400 mg daily; diclofenac was used predominantly in longer duration studies, and naproxen was the predominant comparator in studies of 12 weeks or less. Celecoxib was used in three trials, one comparing celecoxib 400 mg with etoricoxib 90 mg in OA, and two comparing celecoxib 200 mg with etoricoxib 30 mg in OA (Additional file 1). Given the relatively small numbers of patients available, no comparisons were performed on celecoxib data either with placebo or with etoricoxib; with celecoxib there was only about 10% of the number of patients available for NSAID versus placebo, for example.

In OA and RA trials the majority of patients (usually ≥ 70%) were women, and the mean age in the trial was over 60 years. In trials of low back pain, women comprised about 60% of patients, and the mean age was about 50 years. In the trial of ankylosing spondylitis only 20% of patents were women, and the mean age was 44 years. Patients were preponderantly white in all but one trial. One trial (066 MEDAL trial) reported separate data for OA for 60 and 90 mg doses of etoricoxib, and for RA; these separate reporting groups were used in any analysis.

### Discontinuation with placebo over 12 weeks

**Figure 1:**
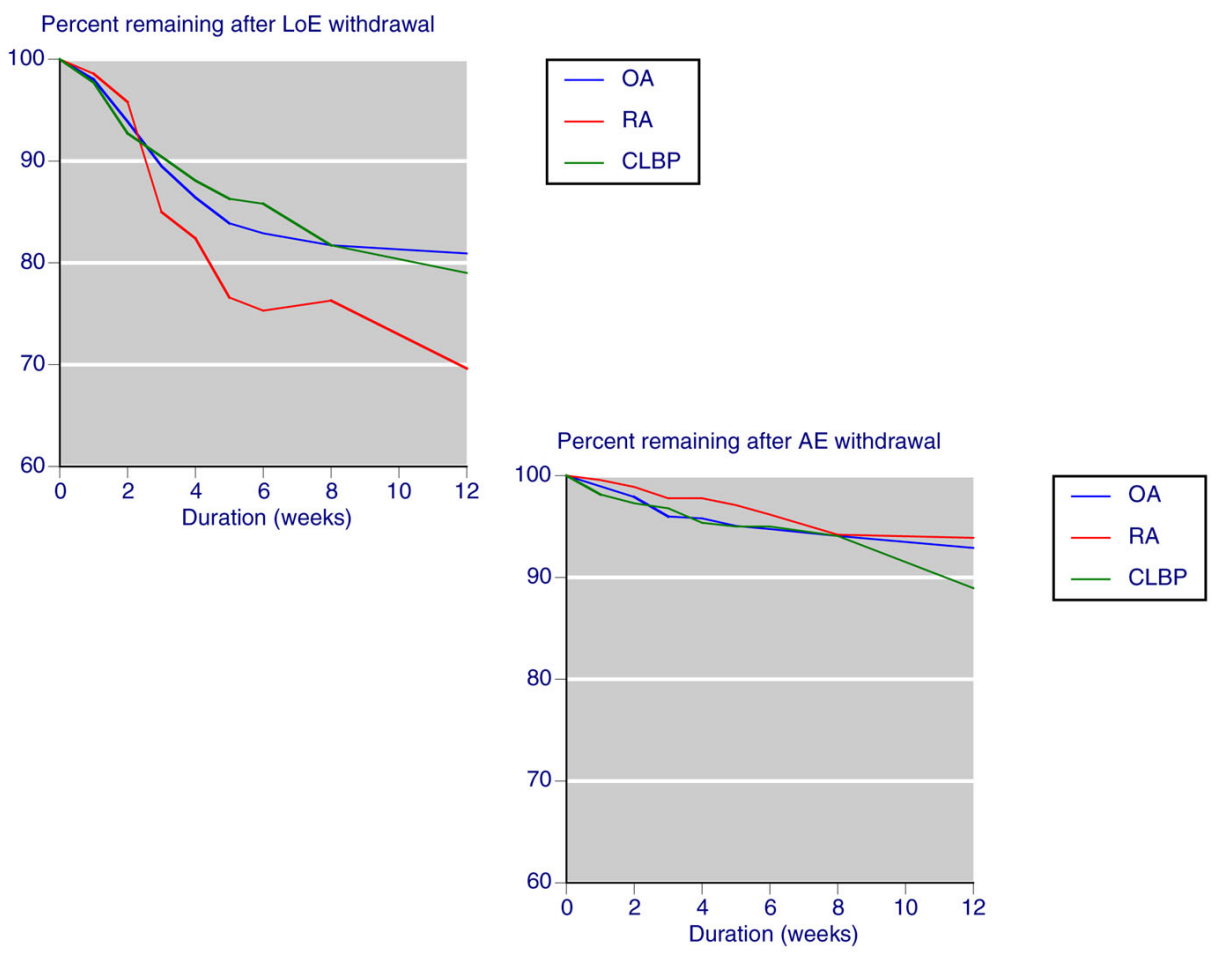
Lack of efficacy or adverse event discontinuation: placebo. Shown are the percentages of patients remaining in the studies conducted over 4 to 12 weeks with placebo after withdrawal because of lack of efficacy (LoE) or an adverse event (AE). CLBP, chronic low back pain; OA, osteoarthritis; RA, rheumatoid arthritis.

Additional files 2 and 3 contain the rates of discontinuation over 12 weeks for any cause and because of lack of efficacy or because of an adverse event, according to whether patients were given placebo or active therapy. Figure 1 shows that discontinuation because of lack of efficacy with placebo tended to be higher in patients with RA than in those with OA or back pain, to the extent of about 10% after 12 weeks. Figure 1 also shows that discontinuation because of adverse events with placebo was not different in patients with different conditions. Overall discontinuation because of lack of efficacy with placebo at 12 weeks was 24%, some three times higher than the percentage of discontinuations because of adverse events.

### Discontinuation with active drug over 12 weeks

**Figure 2:**
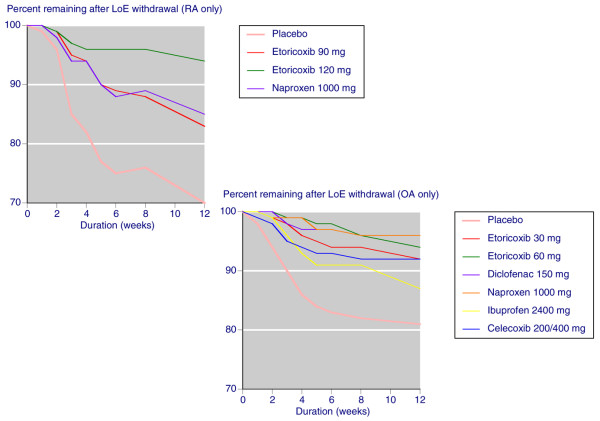
Lack of efficacy discontinuation: placebo, etoricoxib, and individual NSAIDs. Shown are the percentages of patients remaining after lack of efficacy (LoE) discontinuation in studies conducted over 4 to 12 weeks with placebo, etoricoxib, and individual nonsteroidal anti-inflammatory drugs (NSAIDs). OA, osteoarthritis; RA, rheumatoid arthritis.

With active drugs, discontinuation because of lack of efficacy was noticeably less than placebo, with small differences between OA and RA (Figure 2). In OA trials lack of efficacy discontinuation rates at 12 weeks ranged between 4% and 13% for individual drugs and doses, as compared with 19% with placebo. In RA trials discontinuation rates at 12 weeks ranged between 6% and 18% for individual drugs and doses, as compared with 30% with placebo.

**Figure 3:**
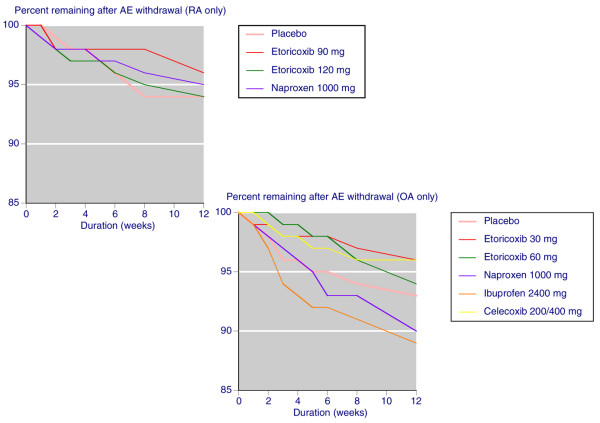
Clinical adverse event discontinuation: placebo, etoricoxib, and individual NSAIDs. Shown are the percentages of patients remaining after clinical adverse event (AE) discontinuation in studies conducted over 4 to 12 weeks with placebo, etoricoxib, and individual nonsteroidal anti-inflammatory drugs (NSAIDs). OA, osteoarthritis; RA, rheumatoid arthritis.

With active drugs, discontinuation because of adverse events was not noticeably different from that with placebo, with small differences between OA and RA (Figure 3). In OA trials discontinuation rates at 12 weeks ranged between 4% and 11% for individual drugs and doses, as compared with 7% with placebo. In RA trials discontinuation rates at 12 weeks ranged between 4% and 6% for individual drugs and doses, as compared with 6% with placebo.

For lack of efficacy discontinuation there was an appreciable dose-response relation for etoricoxib over the range of 30 mg to 120 mg daily for OA and RA (Table 1). For adverse event discontinuation there was no appreciable dose-response relation. For no other condition were there sufficient numbers of trials, patients and doses to make any sensible comparison.

**Table 1:** Dose-response of etoricoxib for lack of efficacy and clinical adverse event discontinuations in osteoarthritis and rheumatoid arthritis trials

Dose etoriocoxib (mg)	Number of patients	Discontinuations (%)	
		
		Lack of efficacy	Clinical adverse event
Osteoarthritis			
0	974	16	5.2
30	1015	7.3	4.1
60	814	3.6	4.8
90	112	1.8	5.4
120	221	0.9	7.7
Rheumatoid arthritis			
0	1,143	29	2.7
60	126	1.6	4.8
90	913	14	2.7
120	463	3.9	5.4

### Etoricoxib discontinuations compared with placebo and active comparators

Table 2 shows the outcomes of patient discontinuations from any cause, because of lack of efficacy, a clinical adverse event, a laboratory adverse event, a gastrointestinal adverse event, serious adverse event, or because of hypertension or oedema. All doses of etoricoxib combined were compared with placebo, and with traditional NSAIDs combined, in trials lasting 4 to 12 weeks, and in all trials combined. These analyses did not include celecoxib as a comparator.

**Table 2:** Analysis of patients with adverse events and discontinuations

Outcome/comparisons	Number of		Rate of events (%)		Relative risk (95% CI)	NNTp/NNH (95% CI)
			
	Trials	Patients	Etoricoxib	Comparator		
All cause						
Etoricoxib versus placebo (4 to 12 weeks)	16	6,388	17	32	0.6 (0.53 to 0.63)^a^	6.7 (5.8 to 7.8)^b^
NSAID versus placebo (4 to 12 weeks)	10	3,690	26	32	0.9 (0.80 to 0.99)^a^	19 (13 to 31)^b^
Etoricoxib versus NSAID (4 to 12 weeks)	12	5,186	17	22	0.7 (0.65 to 0.82)^a^	16 (11 to 29)^b^
Etoricoxib versus NSAID (all trials)	17	39,881	47	51	0.95 (0.93 to 0.97)^a^	31 (24 to 44)^b^
Lack of efficacy						
Etoricoxib versus placebo (4 to 12 weeks)	16	6,388	7.2	22	0.4 (0.32 to 0.41)^a^	7.4 (6.3 to 8.9)^b^
NSAID versus placebo (4 to 12 weeks)	10	3,690	9.1	23	0.5 (0.45 to 0.62)^a^	6.6 (5.9 to 7.6)^b^
Etoricoxib versus NSAID (4 to 12 weeks)	12	5,186	7.6	7.5	1.0 (0.8 to 1.2)	
Etoricoxib versus NSAID (all trials)	17	39,881	8.8	9.5	0.9 (0.86 to 0.97)^a^	145 (80 to 820)^b^
Clinical adverse event						
Etoricoxib versus placebo (4 to 12 weeks)	16	6,388	5.1	4.4	1.2 (0.9 to 1.4)	
NSAID versus placebo (4 to 12 weeks)	10	3,690	8.2	3.8	1.8 (1.4 to 2.4)^a^	23 (17 to 35)^c^
Etoricoxib versus NSAID (4 to 12 weeks)	12	5,186	5	8.2	0.7 (0.53 to 0.81)^a^	31 (22 to 54)^b^
Etoricoxib versus NSAID (all trials)	17	39,881	17	18	1.0 (0.94 to 1.02)	
Laboratory adverse event						
Etoricoxib versus placebo (4 to 12 weeks)	16	6,388	0.7	0.7	0.9 (0.5 to 1.7)	
NSAID versus placebo (4 to 12 weeks)	10	3,690	0.7	0.6	0.9 (0.4 to 2.1)	
Etoricoxib versus NSAID (4 to 12 weeks)	12	5,186	0.3	0.5	0.6 (0.3 to 1.4)	
Etoricoxib versus NSAID (all trials)	17	39,881	1.2	3.3	0.4 (0.34 to 0.45)^a^	49 (43 to 57)^b^
Gastrointestinal adverse event						
Etoricoxib versus placebo (4 to 12 weeks)	16	6,388	1.9	1.7	1.3 (0.9 to 1.8)	
NSAID versus placebo (4 to 12 weeks)	10	3,690	4.7	1.6	2.6 (1.7 to 3.9)	33 (24 to 52)^c^
Etoricoxib versus NSAID (4 to 12 weeks)	12	5,186	2.0	4.1	0.5 (0.38 to 0.73)^a^	47 (33 to 85)^b^
Etoricoxib versus NSAID (all trials)	17	39,881	4.4	7.7	0.6 (0.54 to 0.63)^a^	30 (27 to 35)^b^
Serious adverse event						
Etoricoxib versus placebo (4 to 12 weeks)	16	6,388	0.9	0.7	1.0 (0.6 to 1.7)	
NSAID versus placebo (4 to 12 weeks)	10	3,690	0.9	0.6	1.2 (0.6 to 2.4)	
Etoricoxib versus NSAID (4 to 12 weeks)	12	5,186	0.7	0.9	0.8 (0.5 to 1.6)	
Etoricoxib versus NSAID (all trials)	17	39,881	5.5	5.3	1.1 (0.98 to 1.2)	
Hypertension						
Etoricoxib versus placebo (4 to 12 weeks)	16	6,388	0.4	0.04	1.6 (0.8 to 3.5)	
NSAID versus placebo (4 to 12 weeks)	10	3,690	0.5	0.1	2.0 (0.8 to 4.9)	
Etoricoxib versus NSAID (4 to 12 weeks)	12	5,186	0.5	0.5	1.0 (0.5 to 2.0)	
Etoricoxib versus NSAID (all trials)	17	39,881	2.1	1.3	1.7 (1.4 to 1.9)^a^	120 (94 to 180)^c^
Oedema						
Etoricoxib versus placebo (4 to 12 weeks)	16	6,388	0.3	0.1	1.2 (0.6 to 2.6)	
NSAID versus placebo (4 to 12 weeks)	10	3,690	0.4	0.1	1.6 (0.6 to 4.2)	
Etoricoxib versus NSAID (4 to 12 weeks)	12	5,186	0.3	0.4	1.1 (0.5 to 2.3)	
Etoricoxib versus NSAID (all trials)	17	39,881	1.0	0.7	1.5 (1.2 to 1.9)^a^	320 (200 to 720)^c^

Note that nonsteroidal anti-inflammatory drugs (NSAIDs) indicates traditional NSAIDs only. ^a^Statistically significant difference in relative risk. ^b^Number needed to treat to prevent an event (NNTp). ^c^Number needed to cause an event (NNH). CI, confidence interval.

Etoricoxib and NSAIDs had lower rates of all-cause discontinuation than did placebo (NNTp 6.7 and 19, respectively). Etoricoxib had lower all-cause discontinuations rates than did NSAIDs in studies conducted over 4 to 12 weeks and in those of longer duration (NNTp 16 and 31, respectively). This result was largely driven by lack of efficacy discontinuation, which for placebo constitutes 72% of all discontinuations. Thus, etoricoxib and NSAIDs had lower rates of lack of efficacy discontinuation than did placebo (NNTp 7.4 and 6.6, respectively). Etoricoxib had a lower all-cause discontinuation rate than did NSAIDs in all trials (NNTp 31).

For clinical adverse event discontinuation, NSAIDs were associated with a significantly higher rate than placebo (NNH 23), whereas etoricoxib had significantly fewer clinical adverse event discontinuations than did placebo in studies conducted over 4 to 12 weeks (NNTp 31) but not in trials of longer duration. For laboratory adverse event discontinuations, etoricoxib was associated with significantly fewer than was NSAIDs in trials of longer duration (NNTp 49), with laboratory adverse event discontinuations driven largely by elevated liver enzymes with diclofenac, which is the only NSAID used in the longer duration trials.

**Figure 4:**
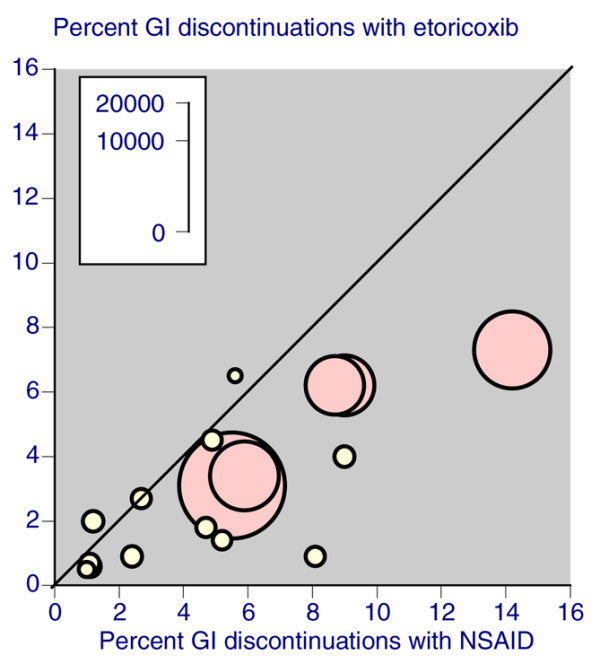
Gastrointestinal discontinuations with etoricoxib and placebo in individual trials. Yellow symbols are trials lasting 4 to 12 weeks; red symbols are trials of longer duration. GI, gastrointestinal; NSAID, nonsteroidal anti-inflammatory drug.

Gastrointestinal adverse event discontinuations were not different between etoricoxib and placebo in trials conducted over 4 to 12 weeks, but they were significantly higher with NSAIDs than with placebo over the same period (NNH 33). Etoricoxib at all doses was associated with significantly fewer gastrointestinal adverse event discontinuations than was NSAIDs in shorter and longer trials (NNTp 47 and 30, respectively). Although the difference between etoricoxib and NSAID tended to be consistent across studies of different duration and size (Figure 4), it was most marked in studies of longer duration.

There was no difference in any comparison for discontinuations because of serious adverse events. Discontinuations because of hypertension and oedema occurred more frequently with etoricoxib than with NSAIDs in trials of longer duration (NNH 120 and 320, respectively).

## Discussion

This analysis demonstrated again that useful and informative information can be obtained from clinical trial reports, which are more detailed in their reporting of the trials than are published papers, if only because they are not constrained by dictates of size. Here, for instance, information from almost 45,000 patients was contained in just over 73,000 pages – close to two pages per patient. This allows for more detail, and the combination of more detail and large numbers of patients offers the possibility of greater insight. This has been seen previously for valdecoxib [15] and celecoxib [16] in arthritis, for efficacy outcomes in acute pain [31,32] and for adverse events for contraceptives [33].

In this case it was possible to examine discontinuation rates and causes in some detail over the first 12 weeks of treatment. With placebo there were slight differences for lack of efficacy discontinuation between conditions, with slightly higher rates for RA over OA, but no difference between conditions for adverse event withdrawals (Figure 1). As would be expected, for placebo lack of efficacy withdrawal at 12 weeks was at least three times higher than was adverse event withdrawal.

With etoricoxib and NSAIDs, lack of efficacy withdrawal was considerably less than with placebo (Table 2 and Figure 2). Etoricoxib was no different from placebo in terms of clinical adverse event discontinuations, but NSAIDs were associated with higher rates, with a NNH of 23. Etoricoxib was associated with lower all-cause discontinuation rates than was placebo, mainly because of fewer lack of efficacy discontinuations. Etoricoxib produced significantly more discontinuations for oedema and hypertension (Table 2).

Additional file 4 shows a wider comparison of discontinuation rates, where data from this analysis are examined alongside data from similar analyses of valdecoxib [15] and celecoxib [16]; information from the valdecoxib and celecoxib analyses is recalculated where necessary to make the comparisons similar. In total, these three analyses involve 81,664 patients enrolled in randomized controlled trials of high quality and validity in the settings of OA, RA, back pain, and ankylosing spondylitis. In 41,608 patients involved in trials lasting 4 to 12 weeks, 6,581 received placebo, 502 paracetamol 4,000 mg daily, 12,093 traditional nonselective NSAIDs at maximum licensed daily doses, and 21,892 received a coxib within the licensed dose range. In trials lasting 26 weeks or longer, 40,596 patients received NSAID at maximum licensed daily doses or a coxib within the licensed dose range. We could find no similar analyses for rofecoxib or lumiracoxib, although the celecoxib analysis provided data on rofecoxib, and the etoricoxib analysis provided information on celecoxib (presented separately in Additional file 4).

**Figure 5:**
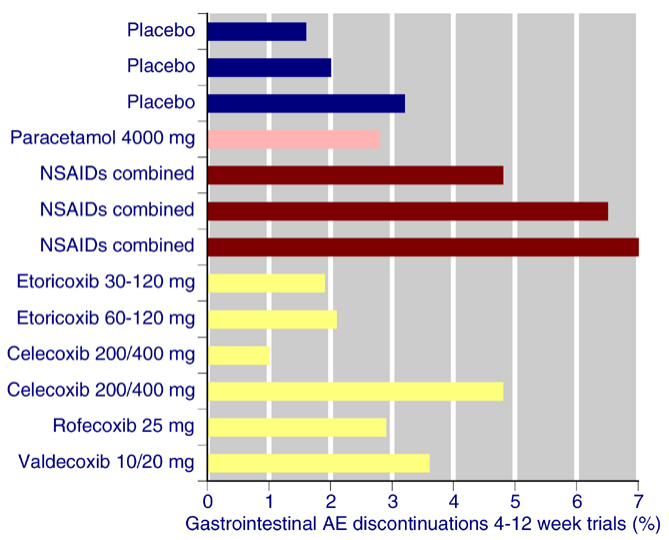
Gastrointestinal AE discontinuations with different therapies in three analyses of trials lasting 4 to 12 weeks. AE, adverse event; NSAID, nonsteroidal anti-inflammatory drug.

This wider comparison shows a degree of consistency between the different datasets. For instance, with placebo the rate of lack of efficacy discontinuation is consistently about 70% of all-cause discontinuations. All-cause discontinuations were lower with NSAIDs (22% to 31%) and paracetamol (25%) than with placebo (32% to 47%), and tended to be lower still with coxibs in trials of 4 to 12 weeks (14% to 31%). Adverse event discontinuations are similar across all treatments: 4.4% to 6.3% with placebo, 5.4% with paracetamol, 5.2% to 8.5% with coxibs, and 7.4% to 11% with NSAIDs. As expected, discontinuations because of a gastrointestinal adverse event tended to be higher with traditional nonselective NSAIDs than with any other treatment (Figure 5). Comparisons in longer duration trials are hampered by a dearth of trials of longer duration using licensed doses of celecoxib and valdecoxib.

Examining discontinuation data from clinical trials, even when the numbers of patients are very large, does not necessarily predict what will happen in the real world, where it is the clinical effectiveness of therapy rather than efficacy in clinical trials that is paramount. There are few comparisons on which to draw, although it is generally assumed that discontinuation rates are higher in clinical practice than in clinical trials. For coxibs and NSAIDs, both clinical trials and observational studies [11,12] concur in their conclusion that adherence is higher with coxibs than with NSAIDs.

Discontinuation results examined here in trials of coxibs and NSAIDs over 12 weeks in musculoskeletal conditions can be compared with trials of other interventions in musculoskeletal and other chronic painful conditions. For example, lack of efficacy and adverse event discontinuations with placebo in short-term trials of opioids in nonmalignant pain were 20% and 7%, respectively [34], similar to our findings for etoricoxib (Table 2). Similar discontinuations rates occur with placebo in 12-week trials in neuropathic pain [35]. Although lack of efficacy discontinuation is typically low (≤ 7%) with active therapies in these conditions, adverse event withdrawal over 12 weeks can be higher (>20% with opioids [34] and about 15% in neuropathic pain), with NNH values for adverse event withdrawal of the order of 10 to 15 [35]. Assessment of adherence in randomized trials is not without its complications and methodological difficulties [36], and this potentially complicates comparisons across datasets.

Discontinuation rates in clinical trials represent important aspects of both efficacy and harm, and deserve more attention. Here, with etoricoxib, discontinuations could be examined in detail because large numbers of patients participated in good quality and well recorded clinical trials, using different doses of etoricoxib and comparators in different clinical conditions. Not all new treatments, and few older ones, enjoy this luxury in terms of the data available.

This does not prevent discontinuations from being a useful measure, perhaps as much or more so in clinical practice as in clinical trials, although discontinuations have been mentioned previously in clinical trials in rheumatology, as with meloxicam in ankylosing spondylitis [37]. In clinical practice, discontinuation rates for adverse events and lack of efficacy has been used to distinguish between disease-modifying antirheumatic drugs [38,39]. Whether discontinuations meet all of the various criteria of truth, discrimination and feasibility that constitute all the possible subquestions in the OMERACT (Outcome Measures in Rheumatology) filter [40] is moot. We know of no formal evaluation, but at face value discontinuation is easy to record, with the cause, and provides useful information.

## Conclusion

This review of a large number of randomized trials and patients provided a more detailed account of discontinuation rates in trials of anti-inflammatory drugs in musculoskeletal conditions than has previously been possible. It also demonstrated consistency between three similar meta-analyses of clinical trial reports covering over 80,000 patients with these conditions. Consistent differences between different drugs were seen, principally fewer adverse event discontinuations with coxibs than with NSAIDs.

## Abbreviations

coxib = cyclo-oxygenase-2 inhibitor; NNH = number needed to treat to cause an event; NNTp = number needed to treat to prevent an event; NSAID = nonsteroidal anti-inflammatory drug; OA = osteoarthritis; RA = rheumatoid arthritis.

## Competing interests

RAM and HJM have received lecture and consultancy fees from pharmaceutical companies with interests in analgesic drugs. The authors have received research support from charities and government sources at various times. This work was supported by an unrestricted educational grant from Merck Inc. The terms of the financial support included freedom for the authors to reach their own conclusions, and an absolute right to publish the results of their research, irrespective of any conclusions reached. Merck did have the right to view the final manuscript before publication, and did so. No author has any direct stock holding in any pharmaceutical company.

## Authors' contributions

RAM was involved in planning the study, data extraction, analysis and preparing a manuscript. SD and HJM were involved in planning, analysis and writing. All authors read and approved the final manuscript.

## Supplementary Material

Additional file 1 Details of the randomized trials included in the review. The file contains information on each included study, with reference, quality score, design, treatments, main results and comments. Click here for file

Additional file 2 Discontinuation rates over 12 weeks because of lack of efficacy in trials conducted over 4 to 12 weeks. The file contains information on percentage of patients not discontinued because of lack of efficacy for weeks 1 to 12, by treatment. Click here for file

Additional file 3 Discontinuation rates over 12 weeks because of adverse events in trials conducted over 4 to 12 weeks. The file contains information on percentage of patients not discontinued because of adverse events for weeks 1 to 12, by treatment. Click here for file

Additional file 4 Comparison between meta-analyses of etoricoxib, valdecoxib, and celecoxib trials. The file contains information on percentage of patients discontinued by end of trial in meta-analyses of etoricoxib, valdecoxib, and celecoxib trials. Click here for file
