# Supplementary material for: Discontinuation rates in clinical trials in musculoskeletal pain: meta-analysis from etoricoxib clinical trial reports
Source: Arthritis Res Ther. 2008 May 8;10(3):R53. doi: 10.1186/ar2422 (PMC2483442; doi:10.1186/ar2422)
Supplement: Additional file 1 — Details of the randomized trials included in the review. The file contains information on each included study, with reference, quality score, design, treatments, main results and comments. [file ar2422-S1.pdf]

Additional fle 1: Details of trials and information extracted on discontinuations

Trial number	Description	Demographics	Duration weeks Compliance Exposure	Treatments (N)	All cause	Lack of efficacy	Clinincal AE
	**Studies of 4-6 weeks**						
805	OA knee or hip<br>At least 40 years<br>Radiographic diagnosis<br>At least 6/12 symptoms<br>Minimum 40/100 mm at inclusion on flare visit, plus ≥15 mm increase and worsening in investigator global assessment since baseline visit	Female 80%<br>Caucasion 81%<br>Mean age 63 years<br>Age range 38-89 years<br>Hip 23%<br>Knee 77%<br>Median duration 5 years<br>ARA Class II/III 80%	6<br>E60 40<br>Diclo 150 41	E60 256<br>Diclo 150 260	E 27<br>Diclo 15	E 7<br>Diclo 1	E 14<br>Diclo 9
806	Chronic low back pain<br>At least 18 years<br>Use of analgesics or NSAID regularly 30 days. Minimum 40/100 mm at inclusion washout visit, plus increase of ≥10 mm, and worsening in investigator assessment since	Female 72%<br>Caucasian 74%<br>Mean age 52 years<br>Age range <20-90 years	4<br>E 60 27<br>Diclo 150 27	E 60 224<br>Diclo 150 222	E 20<br>Diclo 25	E 1<br>Diclo 5	E 15<br>Diclo 13
007	OA knee<br>At least 40 years<br>Radiographic and clinical diagnosis<br>At least 6/12 symptoms<br>Minimum 40/100 mm at inclusion on flare visit, plus ≥15 mm increase and worsening in investigator global assessment since baseline visit	Female 72%<br>White 89%<br>Mean age 61 years<br>Age range 40-87 years<br>Median duration 6 years<br>ARA Class II/III 85%	6<br>E 5 41<br>E10 39<br>E30 42<br>E60 41<br>E90 42<br>Pbo 38	E 5 117<br>E10 114<br>E30 102<br>E60 112<br>E90 112<br>Pbo 60	E 5 12<br>E10 17<br>E30 8<br>E60 9<br>E90 11<br>Pbo 10	E 5 6<br>E10 11<br>E30 4<br>E60 2<br>E90 2<br>Pbo 7	E 5 2<br>E10 2<br>E30 3<br>E60 3<br>E90 6<br>Pbo 2
32	At least 18 years<br>Diagnosis of AS by NY criteria<br>Atleast 6/12 symptoms<br>Minimum 40/100 mm at inclusion flare visit	Female 22%<br>White 71%<br>Mean age 44 years<br>Age range 18-78 years	6<br>E90 41<br>E120 41<br>Nap 1000 39<br>Pbo 31	E90 103<br>E120 92<br>Nap 1000 99<br>Pbo 93	E90 11<br>E120 9<br>Nap 1000 21<br>Pbo 45	E90 8<br>E120 9<br>Nap 1000 20<br>Pbo 44	E90 2<br>E120 0<br>Nap 1000 1<br>Pbo 0

	**Studies of 8-12 weeks**						
010	At least 4 of 7 ARA criteria<br>At least 6/12 duration<br>18 years or older<br>Patient global assessment ≥40 mm, ≥9 tender and ≥6 swollen joints	Female 83%<br>White 62%, Hispanic 27%<br>Mean age 52 years<br>Age range 16 to 82 years<br>Median duration 7 years<br>ARA Class II/III 71%<br>methotrexate 31%,<br>corticosteroid 22%	8<br>E10 52<br>E60 54<br>E90 55<br>E120 53<br>Pbo 53	E10 78<br>E60 126<br>E90 134<br>E120 120<br>Pbo 123	E10 11<br>E60 9<br>E90 6<br>E120 10<br>Pbo 15	E10 5<br>E60 2<br>E90 2<br>E120 3<br>Pbo 11	E10 2<br>E60 6<br>E90 3<br>E120 5<br>Pbo 3
018	OA knee or hip<br>At least 40 years<br>Radiographic diagnosis<br>At least 6/12 symptoms<br>Minimum 40/100 mm at inclusion on flare visit, plus ≥15 mm increase and worsening in investigator global assessment since baseline visit	Female 66%<br>White 91%<br>Mean age 62 years<br>Age range 40 to 92 years<br>Hip 19%<br>Knee 81%<br>Median duration 5 years<br>ARA Class II/III 76%	12<br>E60 78<br>Nap1000 77<br>Pbo 69	E60 222<br>Nap1000 218<br>Pbo 56	E60 37<br>N1000 35<br>Pbo 18	E60 13<br>N1000 9<br>Pbo 11	E60 17<br>N1000 19<br>Pbo 3
019	OA knee or hip<br>At least 40 years<br>Radiographic diagnosis<br>At least 6/12 symptoms<br>Minimum 40/100 mm at inclusion on flare visit, plus ≥15 mm increase and worsening in investigator global assessment since baseline visit	Female 78%<br>White 72%<br>Mean age 63 years<br>Age range 35 to 84 years<br>Hip 25%<br>Knee 75%<br>Median duration 4 years<br>ARA Class II/III 81%	12<br>E60 81<br>Nap1000 77<br>Pbo 73	E60 224<br>Nap1000 221<br>Pbo 56	E60 20<br>N1000 37<br>Pbo 12	E60 7<br>N1000 7<br>Pbo 6	E60 5<br>N1000 24<br>Pbo 6
024	At least 4 of 7 ARA criteria<br>At least 6/12 duration<br>18 years or older<br>Patient global assessment ≥40 mm, ≥6 tender and ≥2 swollen joints	Female 77%<br>White 87%<br>Mean age 56 years<br>Age range 21 to 99 years<br>Median duration 6.7 years<br>ARA Class II/III 79%<br>methotrexate 48%,<br>corticosteroid 32%	12<br>E90 70<br>Nap1000 62<br>Pbo 48	E90 323<br>Nap1000 170<br>Pbo 323	E90 93<br>Nap1000 71<br>Pbo 201	E90 70<br>Nap1000 62<br>Pbo 176	E90 11<br>Nap1000 13<br>Pbo 8

025	At least 4 of 7 ARA criteria<br>At least 6/12 duration<br>18 years or older<br>Patient global assessment ≥40 mm, ≥6 tender and ≥2 swollen joints	Female 77%<br>White 87%<br>Mean age 56 years<br>Age range 21 to 99 years<br>Median duration 6.7 years<br>ARA Class II/III 79%<br>methotrexate 48%, corticosteroid 32%	12<br>E90 76<br>Nap1000 77<br>Pbo 66	E90 353<br>Nap1000 181<br>Pbo 357	E90 59<br>Nap1000 30<br>Pbo 115	E90 44<br>Nap1000 19<br>Pbo 90	E90 9<br>Nap1000 5<br>Pbo 6
026	OA or RA needing NSAID for at least 3 months<br>RA 18 years or older<br>Diagosis at least 6/12<br>OA 50 years or older	Female 82%<br>While 57%, Hispanic 20%<br>Mean age 54 years<br>Age range 18 to 83 years<br>OA 25%, RA 75%	12<br>E120 74<br>Nap1000 69<br>Pbo 75	E120 251<br>Nap1000 244<br>Pbo 247	E120 51<br>Nap1000 94<br>Pbo 42	E120 6<br>Nap1000 3<br>Pbo 14	E120 16<br>Nap1000 25<br>Pbo 14
029	Established OA needing NSAID for at least 3 months<br>At least 50 years	Female 74%<br>White 80%<br>Mean age 62 years<br>Age range 45 to 99 years	12<br>E120 76<br>Ibu 2400 73<br>Pbo 77	E120 221<br>Ibu 2400 226<br>Pbo 233	E120 40<br>Ibu 2400 66<br>Pbo 40	E120 2<br>Ibu 2400 3<br>Pbo 6	E120 17<br>Ibu 2400 15<br>Pbo 11
041	Age 18 to 75 years<br>Mnimum 40 /100 mm at randomisation, >10 mm since screen, worsening since screen	Female 63%<br>White 92%<br>Mean age 53 years<br>Age range 19 to 78 years	12<br>E60 70<br>E90 70<br>Pbo 68	E60 109<br>E90 106<br>Pbo 110	E60 32<br>E90 29<br>Pbo 33	E60 9<br>E90 7<br>Pbo 17	E60 12<br>E90 11<br>Pbo 7

042	Age 18 to 75 years<br>Mnimum 40 /100 mm at randomisation, >10 mm since screen, worsening since screen	Female 61%<br>White 89%<br>Mean age 52 years<br>Age range 20 to 75 years	12<br>E60 71<br>E90 67<br>Pbo 64	E60 103<br>E90 107<br>Pbo 109	E60 28<br>E90 35<br>Pbo 44	E60 6<br>E90 12<br>Pbo 27	E60 12<br>E90 12<br>Pbo 9
065	Age 40 years or more<br>Diagnosis of OA<br>treated with NSAID/coxib	Female 60%<br>White 80%<br>Age range 40 to over 80 years, mean about 60 years	12<br>Drug exposure close to 100%<br>E90 72<br>Cel 400 73<br>Ibu 2400 72<br>Pbo 68	E90 108<br>Cel 400 107<br>Ibu 2400 107<br>Pbo 111	E90 25<br>Cel 400 23<br>Ibu 2400 24<br>Pbo 32	E90 5<br>Cel 400 3<br>Ibu 2400 4<br>Pbo 13	E90 12<br>Cel 400 11<br>Ibu 2400 11<br>Pbo 9
071	OA knee or hip<br>Clinical and radiographic criteria<br>At least 6/12 symptoms<br>Minimum 40/100 mm pain, increase of at least 15 mm from baseline, and worsening since baseline	Female 71%<br>White 89%<br>Mean age 62 years<br>Age range 40 to 89 years	12<br>Compliance 94%<br>Mean days on drug<br>E30 75<br>Ibu 2400 67<br>Pbo 56	E30 214<br>Ibu 2400 210<br>Pbo 104	E30 42<br>Ibu 2400 64<br>Pbo 49	E30 25<br>Ibu 2400 30<br>Pbo 31	E30 11<br>Ibu 2400 22<br>Pbo 6
073	OA knee or hip<br>Clinical and radiographic criteria<br>At least 6/12 symptoms<br>Minimum 40/100 mm pain, increase of at least 15 mm from baseline, and worsening since baseline	Female 76%<br>White 48%<br>Mean age 63 years<br>Age range 41 to 86 years	12<br>Compliance 93%<br>Mean days on drug<br>E30 74<br>Ibu 2400 71<br>Pbo 66	E30 224<br>Ibu 2400 213<br>Pbo 221	E30 41<br>Ibu 2400 46<br>Pbo 32	E30 16<br>Ibu 2400 15<br>Pbo 21	E30 11<br>Ibu 2400 19<br>Pbo 5
076	OA knee or hip<br>Clinical and radiographic criteria<br>At least 6/12 symptoms<br>Minimum 40/100 mm pain, increase of at least 15 mm from baseline, and worsening since baseline	Female 67%<br>White 88%<br>Mean age 62 years<br>Age range 40 to 91 years<br>ARA II/III 82%	12<br>Compliance 99%<br>Mean days on drug<br>E30 78<br>C200 73<br>Pbo 67	E30 231<br>C200 241<br>Pbo 127	E30 40<br>C200 49<br>Pbo 42	E30 14<br>C200 22<br>Pbo 31	E30 10<br>C200 12<br>Pbo 6

077	OA knee or hip<br>Clinical and radiographic criteria<br>At least 6/12 symptoms<br>Minimum 40/100 mm pain, increase of at least 15 mm from baseline, and worsening since baseline	Female 66%<br>White 88%<br>Mean age 62 years<br>Age range 41 to 88 years<br>ARA II/III 79%	12<br>Compliance 99%<br>Mean days on drug<br>E30 79<br>C200 76<br>Pbo 56	E30 244<br>C200 247<br>Pbo 117	E30 32<br>C200 45<br>Pbo 57	E30 15<br>C200 24<br>Pbo 39	E30 7<br>C200 8<br>Pbo 12
	**Studies of >24 weeks**						
061	OA knee, hip, hand, spine<br>Age at least 5 years<br>NSAID needed for 12 months	Female 72%<br>White 85%<br>Mean age 64 years<br>Age range 44 to 92	52<br>E90 9.3<br>Dic 150 8.9<br>E90 2789 py<br>Dic 150 2604 py	E90 3593<br>Dic 150 3518	E90 1455<br>Dic 150 1612	E90 350<br>Dic 150 374	E90 632<br>Dic 150 610
066	At least 50 years<br>OA of knee, hip, hand, or spine or RA (4 of 7 ARA criteria)	Female 74%<br>White 79%<br>Mean age 63 years<br>Age range 45 to 94 years	52+<br>OA<br>E60 20.8 m/11746 py<br>D150 20.2 mo/11288 py<br>OA<br>E90 20.4 mo/3686 py<br>D150 19.4 mo/3491 py<br>RA<br>E90 20.8 mo/4917 py<br>D150 20.1 mo/4786 py	**OA**<br>E60 6769<br>D150 6700<br>**OA**<br>E90 2171<br>D150 2162<br>**RA**<br>E90 2841<br>D150 2855	**OA**<br>E60 3524<br>D150 3590<br>**OA**<br>E90 1584<br>D150 1582<br>**RA**<br>E90 1490<br>D150 1573	**OA**<br>E60 596<br>D150 594<br>**OA**<br>E90 281<br>D150 302<br>**RA**<br>E90 217<br>D150 255	**OA**<br>E60 1133<br>D150 1208<br>**OA**<br>E90 565<br>D150 532<br>**RA**<br>E90 573<br>D150 567
072	At least 50 years<br>RA with at least 4 of 7 ARA criteria	Female 80%<br>White 64%<br>Mean age 61 years<br>Age range 40 to 92 years	52+<br>Compliance 97%<br>E30 19.3 m/3275 py<br>Dic 150 19.1 m/3262 py	E90 2032<br>Dic 150 2054	E90 1028<br>Dic 150 1045	E90 122<br>Dic 150 162	E90 419<br>Dic 150 397

Cause of discontinuations					Time to discontinuation		
**Laboratory AE**	**GI AE**	**Oedema AE**	**Hypertension AE**	**Serious AE**	**All cause**	**Lack of efficacy**	**Adverse event**
E 0<br>Diclo 1	E 5<br>Diclo 3	E 2<br>Diclo 0	E2<br>Diclo 2	E 3<br>Diclo 0	wk E D<br>1 253 252<br>2 251 251<br>3 247 249<br>4 245 241<br>5 243 235<br>6 241 232	wk E D<br>1 256 259<br>2 256 259<br>3 256 255<br>4 255 253<br>5 255 253<br>6 255 253	
E 60 0<br>Diclo 150 0	E 60 6<br>Diclo 150 6	E 60 4<br>Diclo 150 1	E 60 0<br>Diclo 150 0	E 60 1<br>Diclo 150 0	wk E D<br>1 222 218<br>2 213 206<br>3 208 202<br>4 206 198	wk E D<br>1 222 222<br>2 222 215<br>3 222 215<br>4 222 215	wk E D<br>1 222 222<br>2 215 215<br>3 211 213<br>4 208 206
E 5 0<br>E10 1<br>E30 0<br>E60 0<br>E90 0<br>Pbo 0	E 5 0<br>E10 1<br>E30 1<br>E60 1<br>E90 3<br>Pbo 0	E 5 0<br>E10 0<br>E30 0<br>E60 0<br>E90 1<br>Pbo 0	E 5 0<br>E10 0<br>E30 0<br>E60 0<br>E90 0<br>Pbo 0	E 5 2<br>E10 3<br>E30 3<br>E60 4<br>E90 7<br>Pbo 2		wk E30 E60 P<br>1 102 111 59<br>2 101 111 57<br>3 99 111 54<br>4 98 110 53<br>5 98 110 53<br>6 98 110 53	wk E30 E60 P<br>1 102 112 60<br>2 102 110 59<br>3 102 110 59<br>4 101 110 59<br>5 101 109 58<br>6 99 110 58
E90 0<br>E120 0<br>Nap 1000 0<br>Pbo 0	E90 1<br>E120 0<br>Nap 1000<br>1<br>Pbo 0	E90 0<br>E120 0<br>Nap 1000<br>0<br>Pbo 0	E90 0<br>E120 0<br>Nap 1000 0<br>Pbo 0	E90 2<br>E120 0<br>Nap 1000 2<br>Pbo 0		wk E90 E120 N1000 P<br>1 103 91 99 88<br>2 103 91 96 84<br>3 98 86 88 63<br>4 97 85 86 57<br>5 96 84 80 50<br>6 96 83 79 49	wk E90 E120 N1000 P<br>1 103 92 99 93<br>2 102 92 99 93<br>3 102 92 99 93<br>4 102 92 99 93<br>5 101 92 98 93<br>6 101 92 98 93

E10 1	E10 0	E10 0	E10 0	E10 0	wk E60 E90 P	wk E60 E90 P
E60 0	E60 4	E60 1	E60 0	E60 1	1 126 134 122	1 125 133 123
E90 0	E90 0	E90 0	E90 0	E90 3	2 126 134 117	2 123 131 122
E120 0	E120 3	E120 0	E120 0	E120 2	3 126 134 117	3 122 131 122
Pbo 0	Pbo 0	Pbo 0	Pbo 0	Pbo 3	4 126 134 116	4 122 131 122
					5 126 131 113	5 121 131 121
					6 126 131 113	6 121 131 121
					8 123 131 112	8 120 131 119
E60 0	E60 1	E60 0	E60 1	E60 3	wk E60 N1000 P	wk E60 N1000 P
N1000 1	N1000 2	N1000 1	N1000 0	N1000 1	1 222 218 54	1 222 216 56
Pbo 0	Pbo 0	Pbo 1	Pbo 0	Pbo 1	2 222 216 53	2 220 216 56
					3 220 214 49	3 218 214 56
					4 218 214 45	4 218 211 56
					5 213 209 45	5 218 209 55
					6 213 209 45	6 215 207 55
					8 213 209 45	8 211 207 55
					12 209 209 45	12 206 201 53
E60 0	E60 2	E60 0	E60 0	E60 0	wk E60 N1000 P	wk E60 N1000 P
N1000 0	N1000 18	N1000 0	N1000 0	N1000 7	1 224 221 55	1 224 217 54
Pbo 0	Pbo 2	Pbo 0	Pbo 0	Pbo 0	2 224 219 54	2 222 214 54
					3 222 219 53	3 220 212 52
					4 222 219 53	4 220 210 52
					5 222 217 52	5 220 206 52
					6 222 217 51	6 220 203 51
					8 222 214 51	8 220 201 51
					12 220 212 50	12 219 194 49
E90 1	E90 3	E90 1	E90 1	E90 4	wk E90 N1000 P	wk E90 N1000 P
Nap1000 1	Nap1000 4	Nap1000 0	Nap1000 1	Nap1000 1	1 323 170 317	1 320 168 320
Pbo 1	Pbo 4	Pbo 0	Pbo 0	Pbo 2	2 320 165 304	2 317 168 317
					3 297 148 229	3 317 167 313
					4 291 145 213	4 317 165 313
					5 275 128 178	5 313 163 310
					6 271 122 168	6 313 163 307
					8 268 121 162	8 313 163 307
					12 252 107 142	12 310 160 307

E90 0	E90 2	E90 0	E90 0	E90 0
Nap1000 0	Nap1000 2	Nap1000 0	Nap1000 1	Nap1000 1
Pbo 4	Pbo 2	Pbo 0	Pbo 0	Pbo 0

wk	E90	N1000	P
1	349	181	353
2	349	179	343
3	339	176	318
4	335	176	311
5	321	172	289
6	318	170	286
8	314	167	282
12	311	161	261

wk	E90	N1000	P
1	353	179	357
2	349	177	353
3	349	177	350
4	349	177	350
5	346	177	346
6	346	177	346
8	346	176	343
12	342	176	343

E120 0	E120 10	E120 0	E120 0	E120 2
Nap1000 0	Nap1000 22	Nap1000 0	Nap1000 1	Nap1000 1
Pbo 1	Pbo 1	Pbo 0	Pbo 0	Pbo 6

wk	E120	N1000	P
1	251	242	247
2	248	242	247
3	246	242	245
4	243	242	245
5	243	242	245
6	243	242	245
8	238	239	245
12	236	239	242

wk	E120	N1000	P
1	248	242	245
2	243	239	245
3	243	237	240
4	243	237	240
5	241	234	240
6	238	232	232
8	238	232	220
12	236	229	220

E120 2	E120 10	E120 1	E120 2	E120 2
Ibu 2400 0	Ibu 2400 11	Ibu 2400 1	Ibu 2400 0	Ibu 2400 3
Pbo 0	Pbo 9	Pbo 0	Pbo 0	Pbo 2

wk	E120	Ibu2400	P
1	221	226	233
2	221	226	231
3	221	226	231
4	221	226	231
5	221	226	231
6	221	224	228
8	219	224	228
12	219	221	226

wk	E120	Ibu2400	P
1	219	226	233
2	219	224	231
3	217	221	228
4	212	221	228
5	212	221	226
6	208	219	226
8	203	215	224
12	201	210	221

E60 2	E60 4	E60 0	E60 0	E60 0
E90 3	E90 3	E90 1	E90 0	E90 1
Pbo 2	Pbo 1	Pbo 0	Pbo 0	Pbo 0

wk	E60	E90	P
1	105	104	107
2	103	104	104
3	102	104	101
4	102	104	97
5	99	102	97
6	99	102	97
8	98	101	93
12	96	99	92

wk	E60	E90	P
1	105	104	108
2	104	98	106
3	102	96	105
4	101	94	102
5	98	93	102
6	96	93	102
8	96	92	101
12	91	91	99

E60 2	E60 1	E60 2	E60 1	E60 1
E90 5	E90 4	E90 1	E90 2	E90 1
Pbo 1	Pbo 4	Pbo 1	Pbo 0	Pbo 1

wk	E60	E90	P
1	103	106	107
2	100	105	99
3	100	100	97
4	100	99	96
5	99	99	92
6	99	98	91
8	98	96	86
12	97	95	81

wk	E60	E90	P
1	101	107	107
2	97	104	107
3	94	101	107
4	93	100	107
5	93	96	106
6	92	95	106
8	92	93	105
12	89	89	96

E90 0	E90 7	E90 1	E90 2	E90 1
Cel 400 0	Cel 400 2	Cel 400 0	Cel 400 0	Cel 400 3
Ibu 2400 0	Ibu 2400 6	Ibu 2400 1	Ibu 2400 0	Ibu 2400 0
Pbo 0	Pbo 0	Pbo 0	Pbo 0	Pbo 0

wk	E90	C400	I2400	P
6	105	105	103	101
12	103	104	103	98

wk	E90	C400	I2400	P
6	102	98	98	103
12	96	96	96	102

E30 0	E30 3	E30 0	E30 2	E30 2
Ibu 2400 2	Ibu 2400 11	Ibu 2400 3	Ibu 2400 3	Ibu 2400 2
Pbo 0	Pbo 2	Pbo 0	Pbo 1	Pbo 0

wk	E30	Ibu2400	P
1	212	208	102
2	210	206	95
3	208	197	86
4	203	191	81
5	199	187	75
6	197	187	74
8	195	185	73
12	188	176	71

wk	E30	Ibu2400	P
1	214	206	103
2	212	204	102
3	210	200	102
4	210	197	102
5	208	195	101
6	208	195	99
8	208	189	97
12	201	183	96

E30 0	E30 4	E30 1	E30 2	E30 0
Ibu 2400 2	Ibu 2400 10	Ibu 2400 1	Ibu 2400 2	Ibu 2400 0
Pbo 2	Pbo 8	Pbo 0	Pbo 0	Pbo 0

wk	E30	Ibu2400	P
1	222	213	219
2	222	211	206
3	220	207	197
4	215	204	192
5	211	200	181
6	211	200	179
8	208	198	177
12	204	194	177

wk	E30	Ibu2400	P
1	220	211	221
2	217	207	217
3	217	198	208
4	217	196	208
5	217	194	206
6	217	194	206
8	215	194	206
12	213	192	206

E30 1	E30 3	E30 1	E30 2	E30 1
C200 2	C200 2	C200 1	C200 0	C200 2
Pbo 0	Pbo 0	Pbo 0	Pbo 0	Pbo 1

wk	E30	C200	P
1	231	236	124
2	229	234	118
3	226	231	110
4	226	229	104
5	224	224	98
6	224	224	97
8	224	222	95
12	217	222	95

wk	E30	C200	P
1	226	239	127
2	226	236	126
3	226	234	124
4	226	231	122
5	224	231	122
6	224	231	122
8	224	229	122
12	219	227	121

E30 0<br>C200 1<br>Pbo 0	E30 3<br>C200 2<br>Pbo 5	E30 1<br>C200 0<br>Pbo 0	E30 0<br>C200 0<br>Pbo 0	E30 0<br>C200 2<br>Pbo 3		wk E30 C200 P<br>1 244 247 109<br>2 242 245 101<br>3 239 235 92<br>4 237 232 83<br>5 232 230 82<br>6 229 230 80<br>8 229 227 78<br>12 229 225 76	wk E30 C200 P<br>1 244 247 110<br>2 244 247 109<br>3 242 245 106<br>4 242 245 106<br>5 242 240 106<br>6 242 240 106<br>8 239 240 105<br>12 239 240 103
E90 23<br>Dic 150 194	E90 262<br>Dic 150<br>498	E90 33<br>Dic 150 26	E90 81<br>Dic 150 23	E90 109<br>Dic 150 104	No data	data unextractable	no data
**OA**<br>E60 86<br>D150 175<br>**OA**<br>E90 61<br>D150 135<br>**RA**<br>E90 48<br>D150 81	**OA**<br>E60 213<br>D150 369<br>**OA**<br>E90 134<br>D150 195<br>**RA**<br>E90 96<br>D150 169	**OA**<br>E60 56<br>D150 49<br>**OA**<br>E90 41<br>D150 17<br>**RA**<br>E90 28<br>D150 16	**OA**<br>E60 146<br>D150 109<br>**OA**<br>E90 55<br>D150 24<br>**RA**<br>E90 69<br>D150 46	**OA**<br>E60 404<br>D150 400<br>**OA**<br>E90 201<br>D150 166<br>**RA**<br>E90 243<br>D150 196	Overall treament group all cause discontinuation - plus dease and dose	No data	Data on clinical plus laboratory only
E90 23<br>Dic 150 44	E90 125<br>Dic 150<br>179	E90 23<br>Dic 150 8	E90 51<br>Dic 150 31	E90 129<br>Dic 150 127	No data	data unextractable	data unextractable
