# Supplementary material for: Discontinuation rates in clinical trials in musculoskeletal pain: meta-analysis from etoricoxib clinical trial reports
Source: Arthritis Res Ther. 2008 May 8;10(3):R53. doi: 10.1186/ar2422 (PMC2483442; doi:10.1186/ar2422)
Supplement: Additional file 2 — Discontinuation rates over 12 weeks because of lack of efficacy in trials conducted over 4 to 12 weeks. The file contains information on percentage of patients not discontinued because of lack of efficacy for weeks 1 to 12, by treatment. [file ar2422-S2.pdf]

Additional file 2: Continuation rates (percent remaining in the study) over 12 weeks because of lack of efficacy in trials of 4-12 weeks

All trials (osteoarthritis, rheumatoid arthritis, chronic low back pain, ankylosing spondylitis)

Week	Placebo	Etoricoxib				Diclofenac 150 mg	Naproxen 1000 mg	Ibuprofen 2400 mg	Celecoxib 200/400 mg
		30 mg	60 mg	90 mg	120 mg				
**Number**	**2336**	**1015**	**1376**	**1238**	**664**	**482**	**1113**	**649**	**488**
0	100	100	100	100	100	100	100	100	100
1	98	100	99	99	100	100	100	100	99
2	95	99	99	99	99	98	99	99	98
3	87	98	99	95	98	98	96	97	95
4	85	96	98	95	98	97	95	96	94
5	81	95	98	92	97	97	92	94	93
6	79	94	97	91	97	97	92	94	93
8	79	94	96	89	97		92	94	92
12	76	92	95	85	96		90	91	92

OA trials only

Week	Placebo	Etoricoxib				Diclofenac 150 mg	Naproxen 1000 mg	Ibuprofen 2400 mg	Celecoxib 200/400 mg
		30 mg	60 mg	90 mg	120 mg				
**Number**	**974**	**1015**	**590**			**260**	**439**	**423**	**488**
0	100	100	100			100	100	100	100
1	98	100	100			100	100	100	99
2	94	99	100			100	99	99	98
3	90	98	99			98	99	96	95
4	86	96	99			97	99	93	94
5	84	95	98			97	97	91	93
6	83	94	98			97	97	91	93
8	82	94	96				96	91	92
12	81	92	94				96	87	92

RA trials only

Week	Placebo	Etoricoxib				Diclofenac 150 mg	Naproxen 1000 mg	Ibuprofen 2400 mg	Celecoxib 200/400 mg
		30 mg	60 mg	90 mg	120 mg				
**Number**	**1050**			**810**	**371**		**595**		
0	100			100	100		100		
1	99			100	100		100		
2	96			99	99		98		
3	87			95	98		95		
4	84			94	97		95		
5	79			90	97		91		
6	77			89	97		90		
8	76			88	96		89		
12	70			82	94		85		
