# Supplementary material for: Discontinuation rates in clinical trials in musculoskeletal pain: meta-analysis from etoricoxib clinical trial reports
Source: Arthritis Res Ther. 2008 May 8;10(3):R53. doi: 10.1186/ar2422 (PMC2483442; doi:10.1186/ar2422)
Supplement: Additional file 3 — Discontinuation rates over 12 weeks because of adverse events in trials conducted over 4 to 12 weeks. The file contains information on percentage of patients not discontinued because of adverse events for weeks 1 to 12, by treatment. [file ar2422-S3.pdf]

Additional file 3: Continuation rates (percent remaining in the study) over 12 weeks because of adverse event in trials lasting 4-12 weeks

All trials (osteoarthritis, rheumatoid arthritis, chronic low back pain, ankylosing spondylitis)

		Etoricoxib							
**Week**	**Placebo**	**30 mg**	**60 mg**	**90 mg**	**120 mg**	**Diclofenac 150 mg**	**Naproxen 1000 mg**	**Ibuprofen 2400 mg**	**Celecoxib 200/400 mg**
**Number**	**2336**	**1015**	**1120**	**1238**	**664**		**1113**	**649**	**488**
0	100	100	100	100	100		100	100	100
1	99	99	99	100	99		99	99	100
2	98	99	98	98	98		98	98	99
3	97	98	96	98	98		98	95	98
4	97	98	96	97	97		97	95	98
5	96	98	96	96	96		96	94	97
6	95	98	95	96	95		95	94	97
8	94	97	94	95	94		95	92	96
12	93	96	92	94	93		93	90	96

OA trials only

		Etoricoxib							
**Week**	**Placebo**	**30 mg**	**60 mg**	**90 mg**	**120 mg**	**Diclofenac 150 mg**	**Naproxen 1000 mg**	**Ibuprofen 2400 mg**	**Celecoxib 200/400 mg**
**Number**	**974**	**1015**	**590**				**439**	**423**	**488**
0	100	100	100	100	100		100	100	100
1	99	99	100				99	99	100
2	98	99	99				98	97	99
3	96	98	98				97	94	98
4	96	98	98				96	93	98
5	95	98	98				95	92	97
6	95	98	97				93	92	97
8	94	97	95				93	91	96
12	93	96	93				90	89	96

RA trials only

		Etoricoxib							
**Week**	**Placebo**	**30 mg**	**60 mg**	**90 mg**	**120 mg**	**Diclofenac 150 mg**	**Naproxen 1000 mg**	**Ibuprofen 2400 mg**	**Celecoxib 200/400 mg**
**Number**	**1050**			**810**	**371**		**595**		
0	100	100	100	100	100	100	100	100	100
1	100			100	99		99		
2	99			98	98		98		
3	98			98	97		98		
4	98			98	97		97		
5	97			98	96		96		
6	96			98	95		96		
8	94			98	95		96		
12	94			96	94		95		
