# Supplementary material for: Discontinuation rates in clinical trials in musculoskeletal pain: meta-analysis from etoricoxib clinical trial reports
Source: Arthritis Res Ther. 2008 May 8;10(3):R53. doi: 10.1186/ar2422 (PMC2483442; doi:10.1186/ar2422)
Supplement: Additional file 4 — Comparison between meta-analyses of etoricoxib, valdecoxib, and celecoxib trials. The file contains information on percentage of patients discontinued by end of trial in meta-analyses of etoricoxib, valdecoxib, and celecoxib trials. [file ar2422-S4.pdf]

Additional file 4: Comparison between meta-analyses of etoricoxib, valdecoxib, and celecoxib trials

Outcome	Placebo	Placebo	Placebo	Paracetamol 4000 mg	NSAIDs combined	NSAIDs combined	NSAIDs combined	Etoricoxib 30-120 mg	Celecoxib 200/400 mg	Celecoxib 200/400 mg	Rofecoxib 25 mg	Valdecoxib 10/20 mg
**Origin of data**	E	C	V	C	E	C	V	E	E	C	C	V
**Trials lasting 4-12 weeks**												
**Number of patients**	2447	2990	1144	502	2371	8375	1347	4421	595	13586	1323	1967
**Adverse events**												
At least one adverse event	42	48	50	32	52	49	63	50	45	44	49	56
Serious adverse event	1.6	1.4	2.4	0.5	1.6	2.5	2.1	1.6	2.5	2.5	2.1	1.7
**Discontinuations**												
All cause	32	40	47	25	22	23	31	16	20	23	14	31
Lack of efficacy	22	28	33	11	7.5	6.3	13	6.6	8.2	8.1	1.5	17
Clinical adverse event	4.4	5.5	6.3	5.4	7.4	9.5	11	5.2	5.2	8.5	6.8	6.7
Gastrointestinal adverse event	1.6	2.0	3.2	2.8	4.8	6.5	7.0	1.9	1.0	4.8	2.9	3.6
Serious adverse event	0.7				0.7			0.9	1.2			
**Trials lasting 26 weeks or more**												
**Number of patients**					17289	4310	499	17406		326		766
**Adverse events**												
At least one adverse event					77		69	79				60
Serious adverse event					16		11	17				5.0
**Discontinuations**												
All cause					54	57	31	52		21		25
Lack of efficacy					9.8	18	8.2	9.0		8.0		9.0
Clinical adverse event					19	24	15	19		10		7.8
Gastrointestinal adverse event					8.2	15	11	4.8				3.7
Serious adverse event					5.9			6.2				

Note superscript refers to data set; E= etoricoxib; C = celecoxib; V=valdecoxib
